# Supplementary figures and images for: The Aurora kinase inhibitor AT9283 inhibits Burkitt lymphoma growth by regulating Warburg effect
Source: PeerJ. 2023 Dec 11;11:e16581. doi: 10.7717/peerj.16581 (PMC10720464; doi:10.7717/peerj.16581)

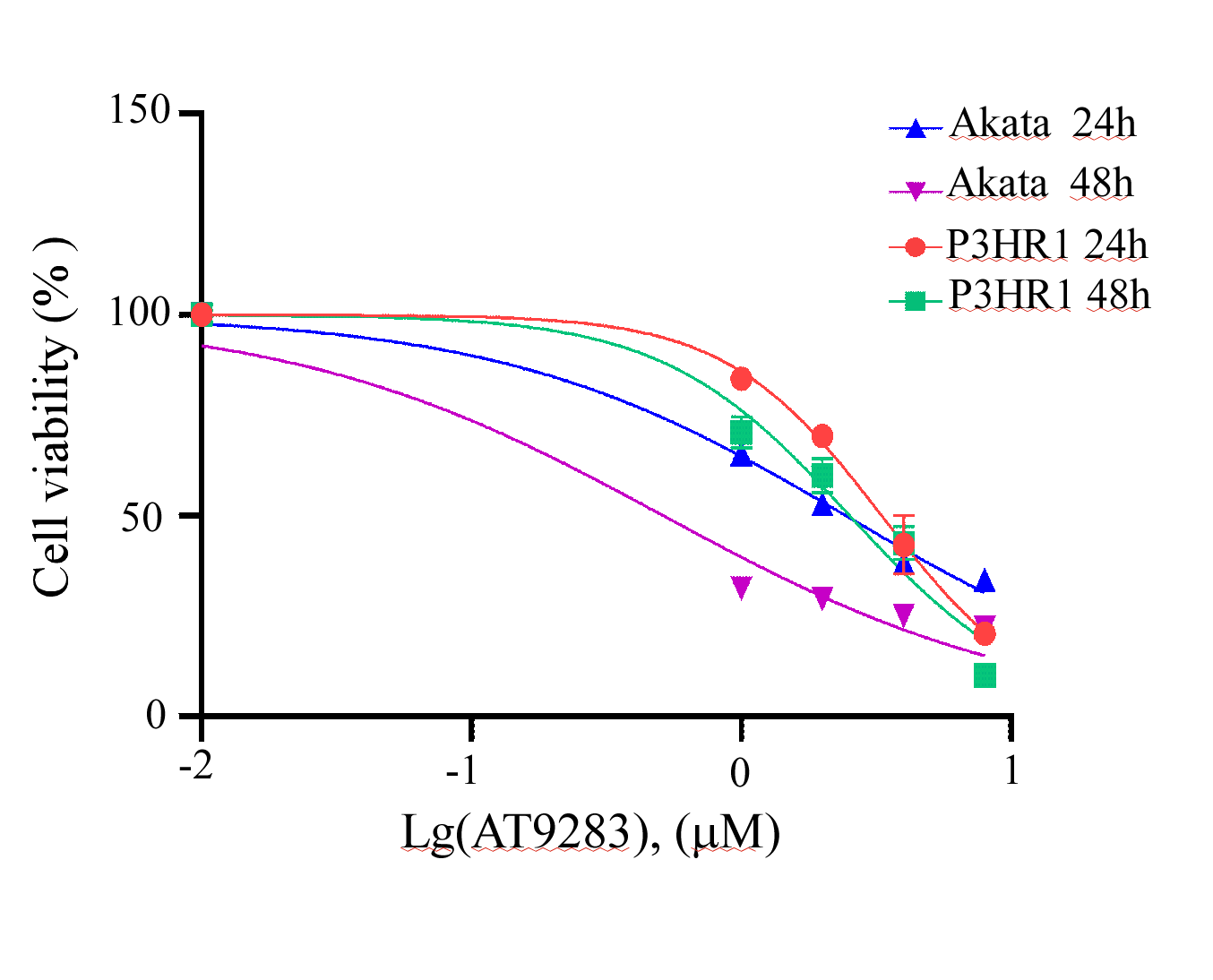

Supplement: Supplemental Information 1 — The dose–response curves of AT9283 in Akata and in P3HR1 cells were calculated using GraphPad sofware with the nonlinear regression curve fit. [file peerj-11-16581-s001.png]

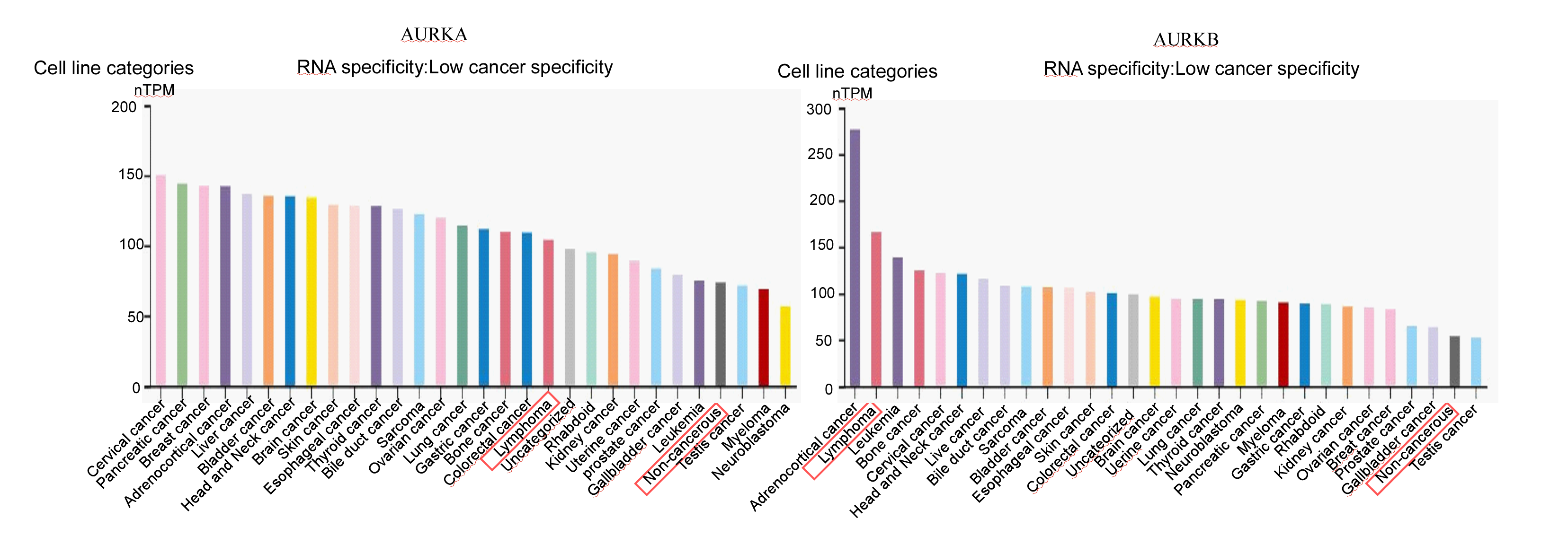

Supplement: Supplemental Information 2 — Using the Human Protein Atlas (HPA, https://www.proteinatlas.org/) database, we evaluated the Aurora A and B mRNA levels in different cell lines. [file peerj-11-16581-s002.png]

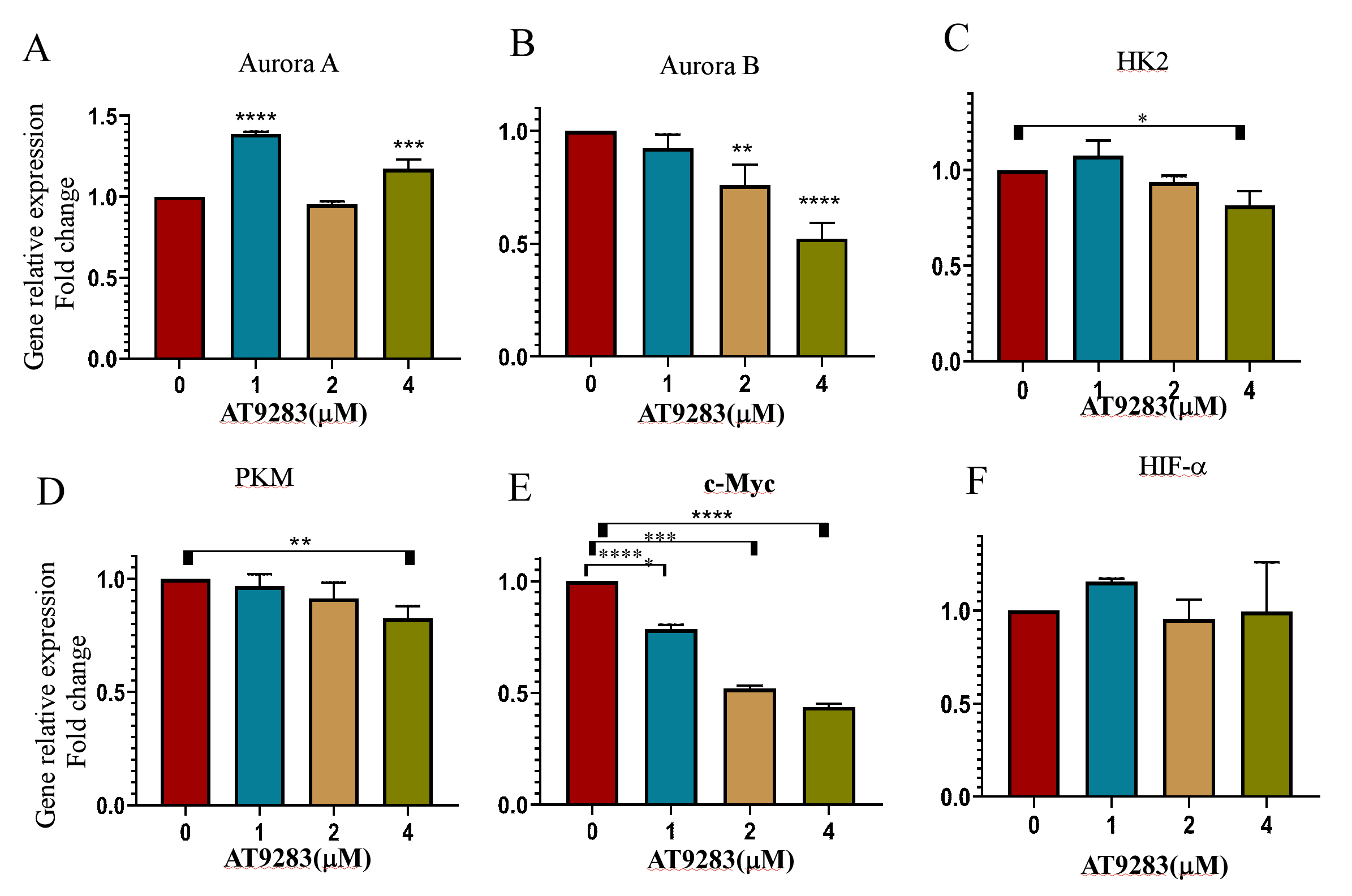

Supplement: Supplemental Information 3 — The cells were cultured with different concentrations of AT9283 for 48 h. Real-time PCR was performed to determine the mRNA level of related genes. The relative level was normalized to the value of β-actin. The results are expressed as fold changes compared to the control. The data represent the mean ± SD of three independent experiments performed in triplicate. *p < 0.05, **p < 0.01, ***p < 0.001 and ****p < 0.0001. [file peerj-11-16581-s003.png]

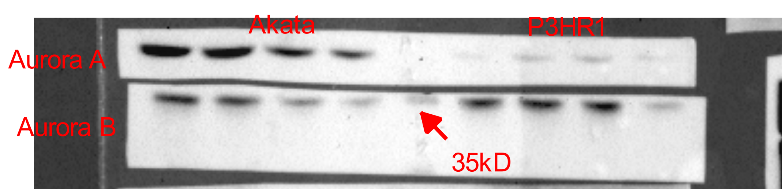


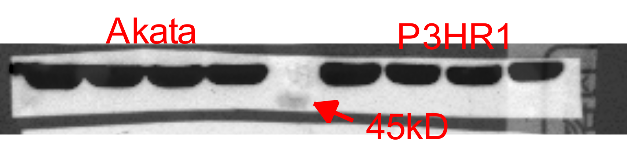


B-actin

Aurora A


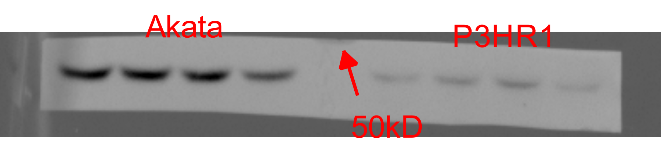

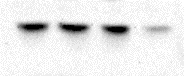

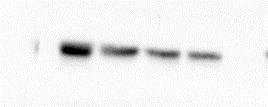


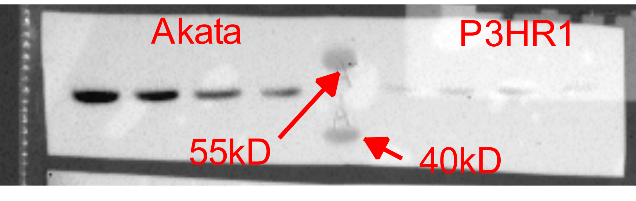


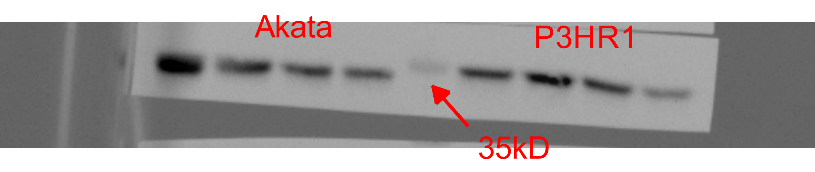
Aurora B


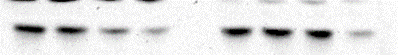


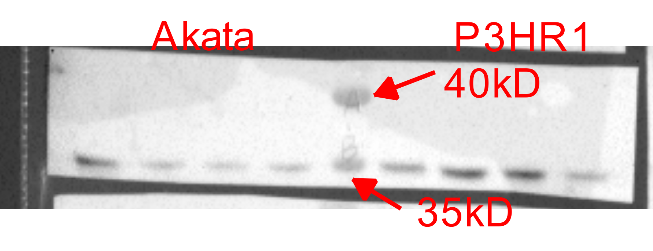


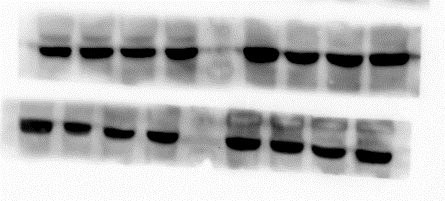


B-actin


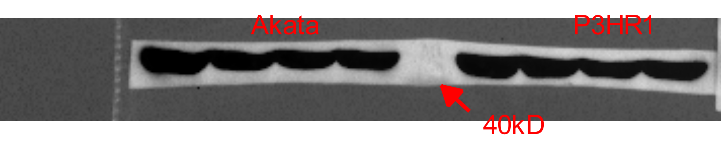


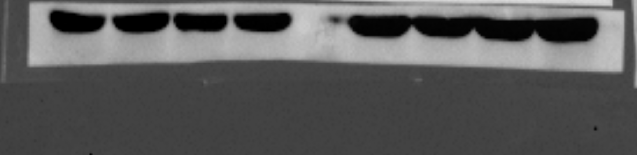

Supplement: Supplemental Information 4 [file peerj-11-16581-s004.docx]

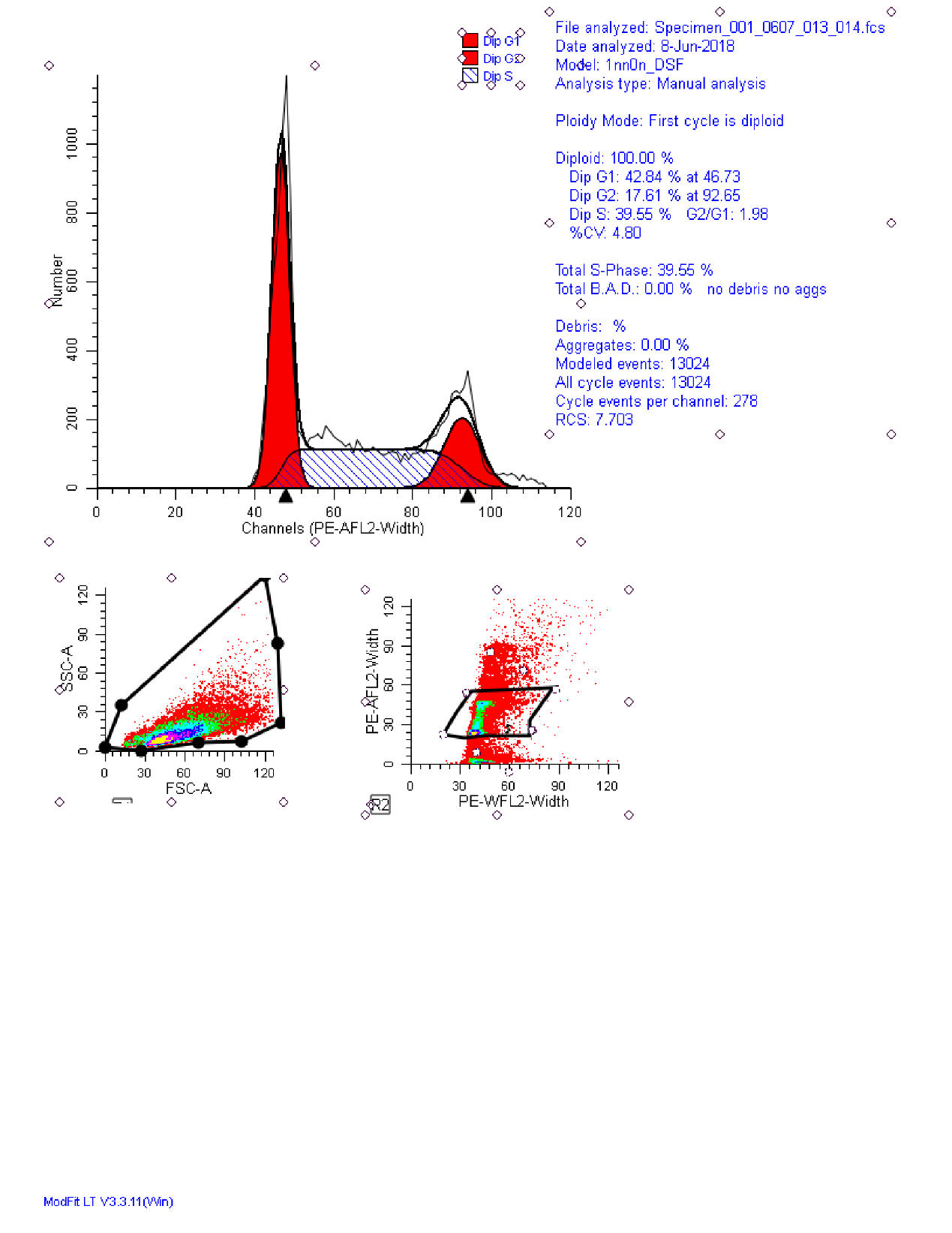
 Figure2

A


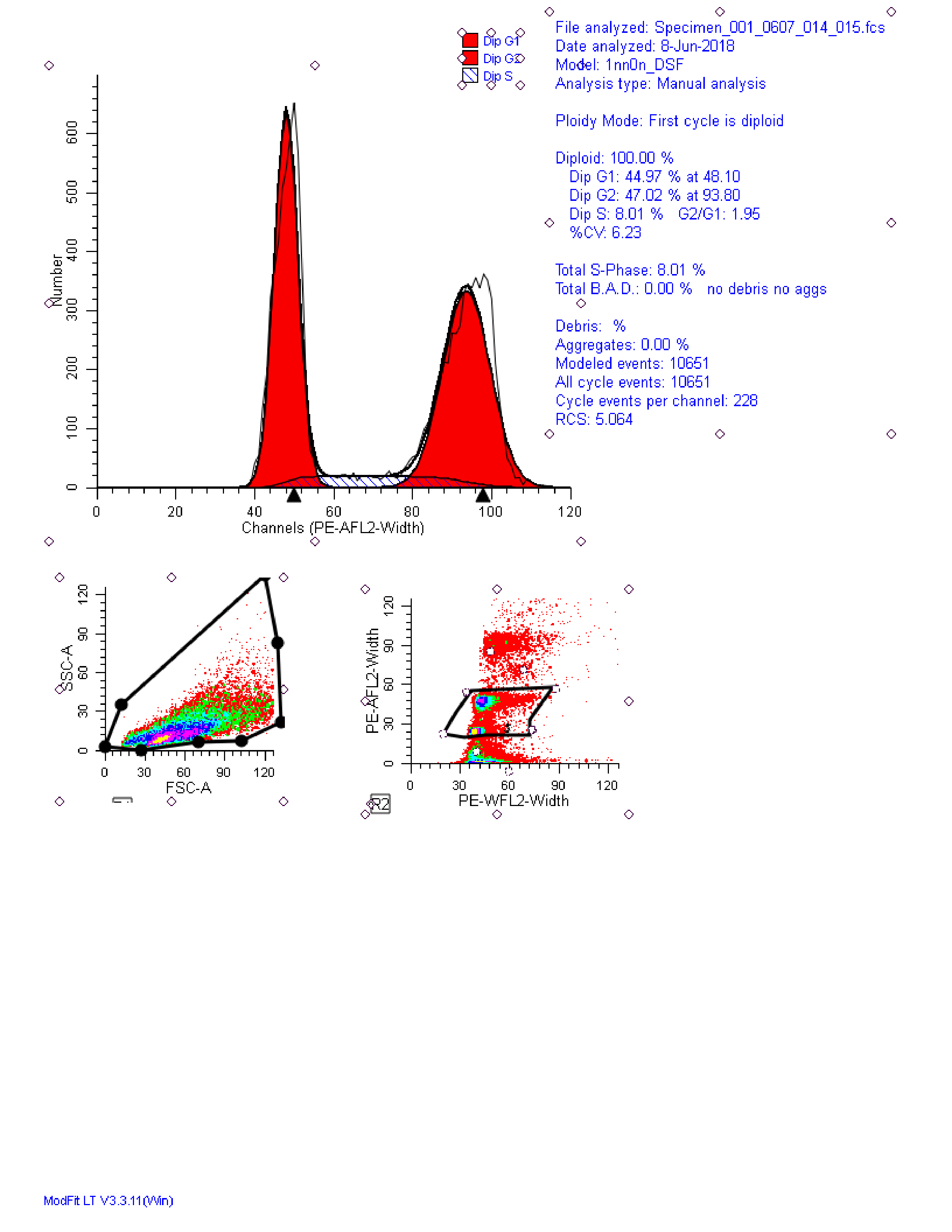

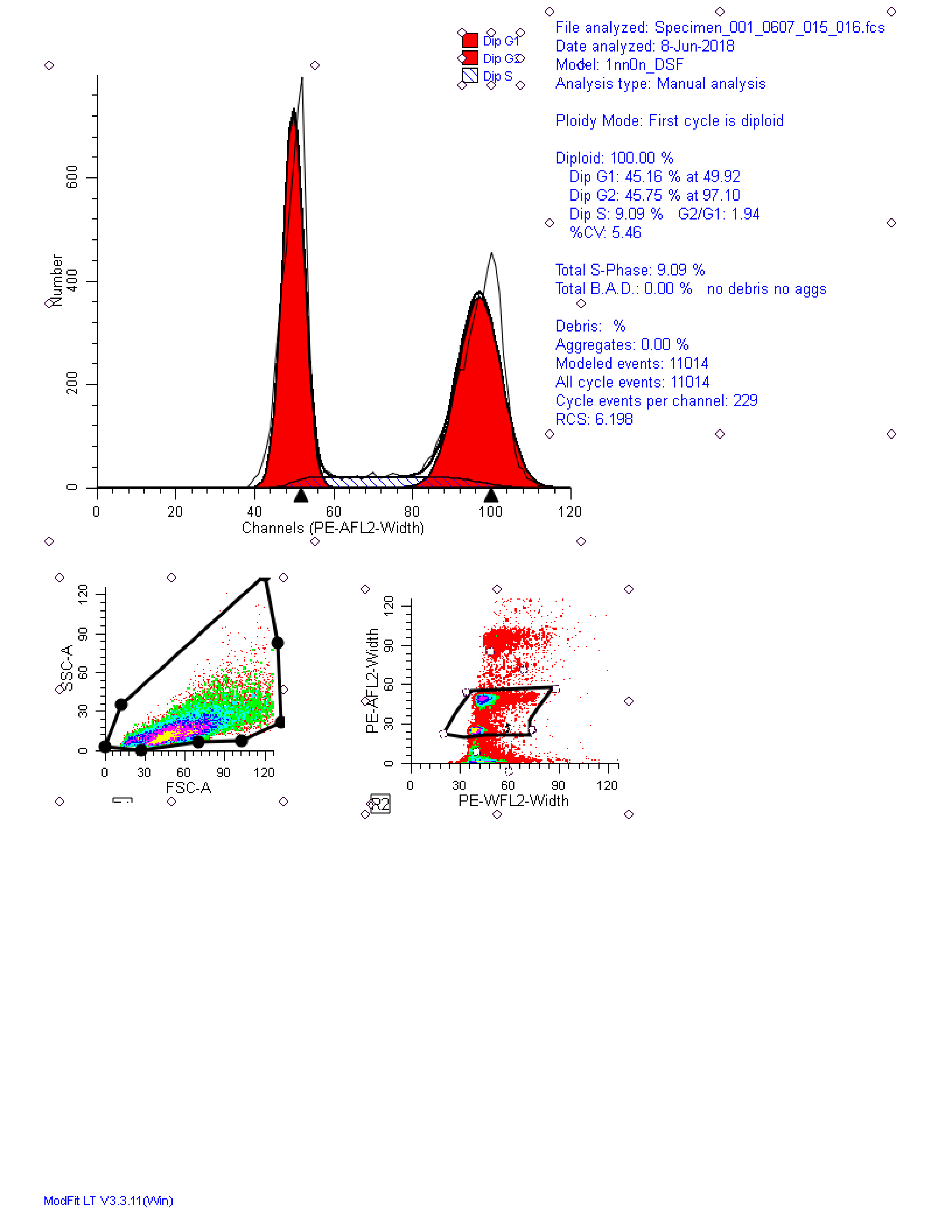

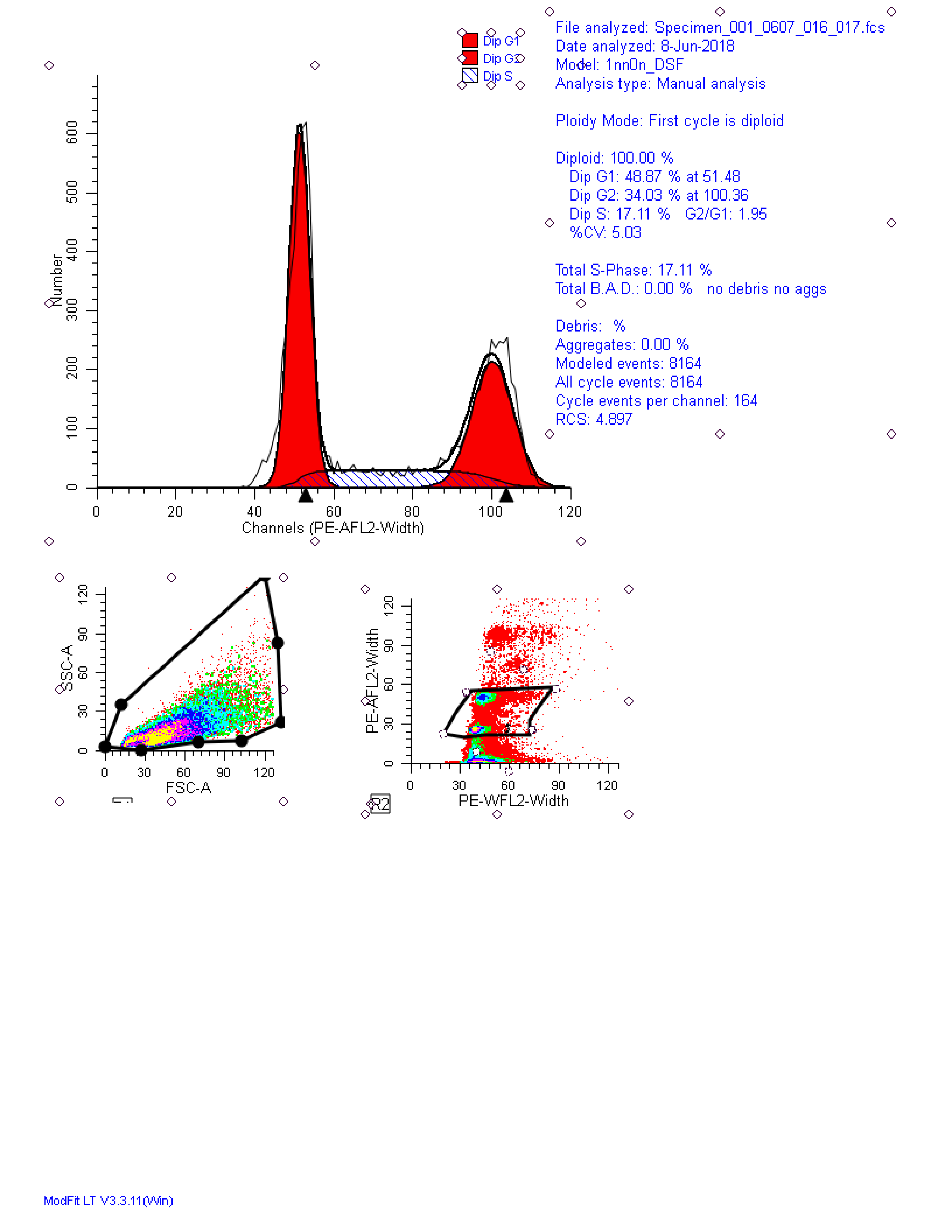

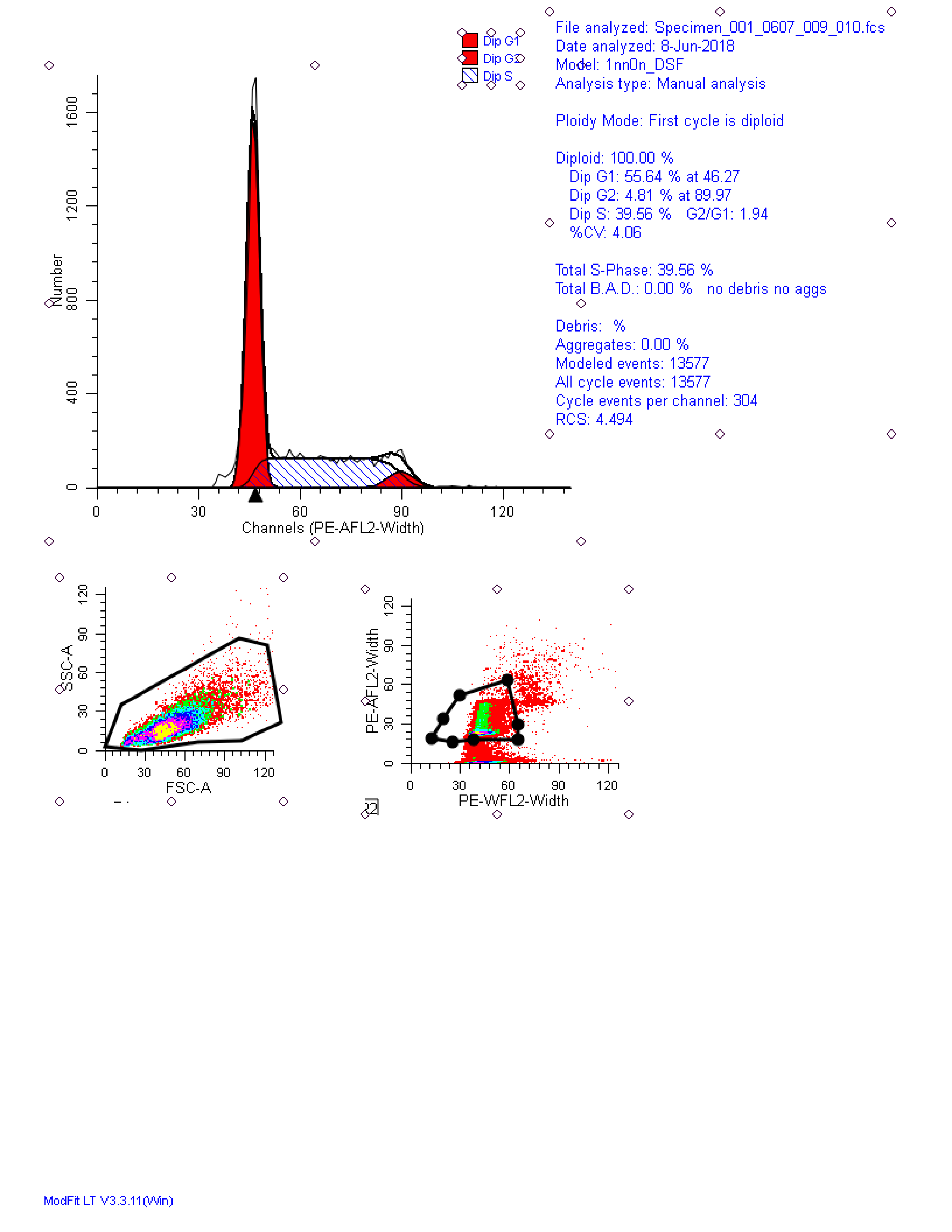

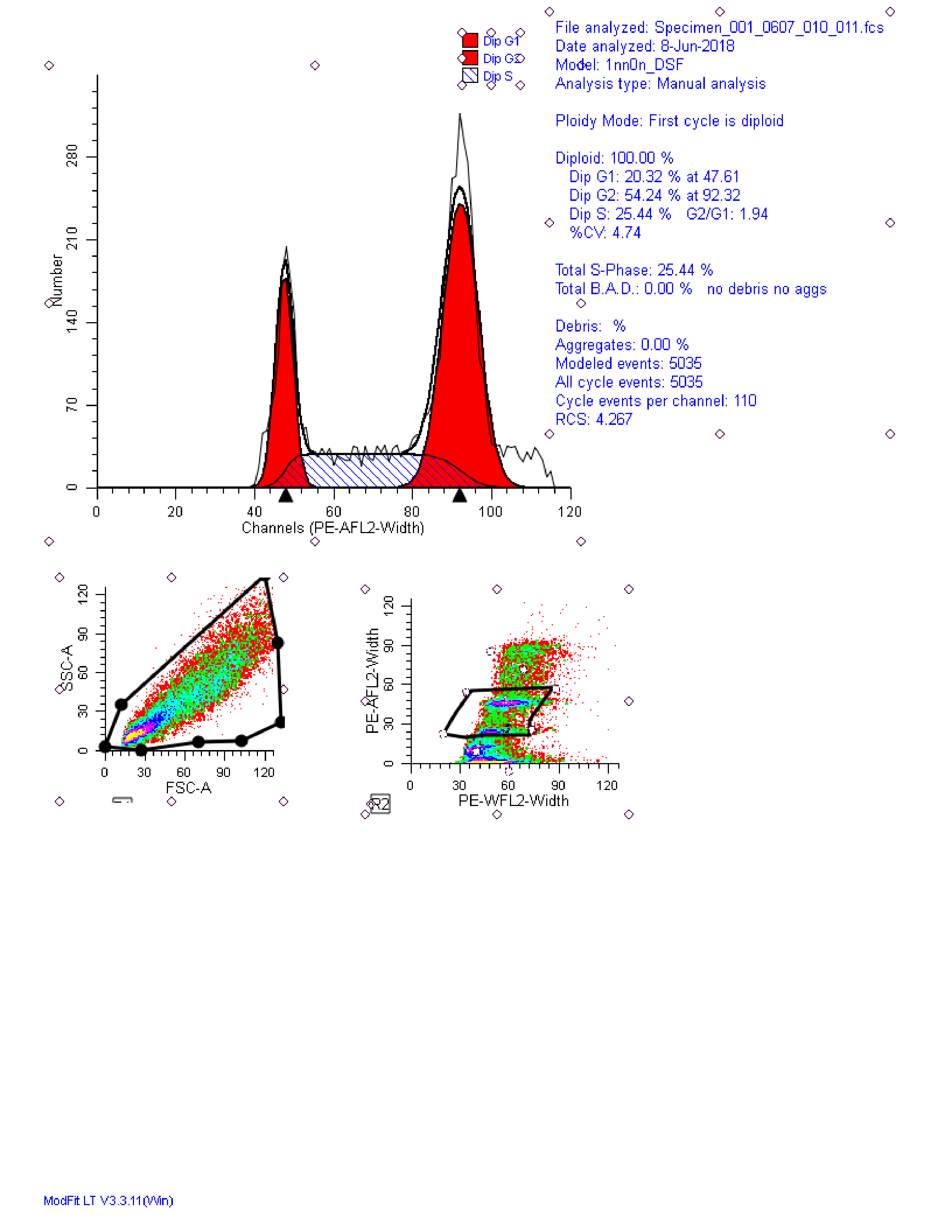

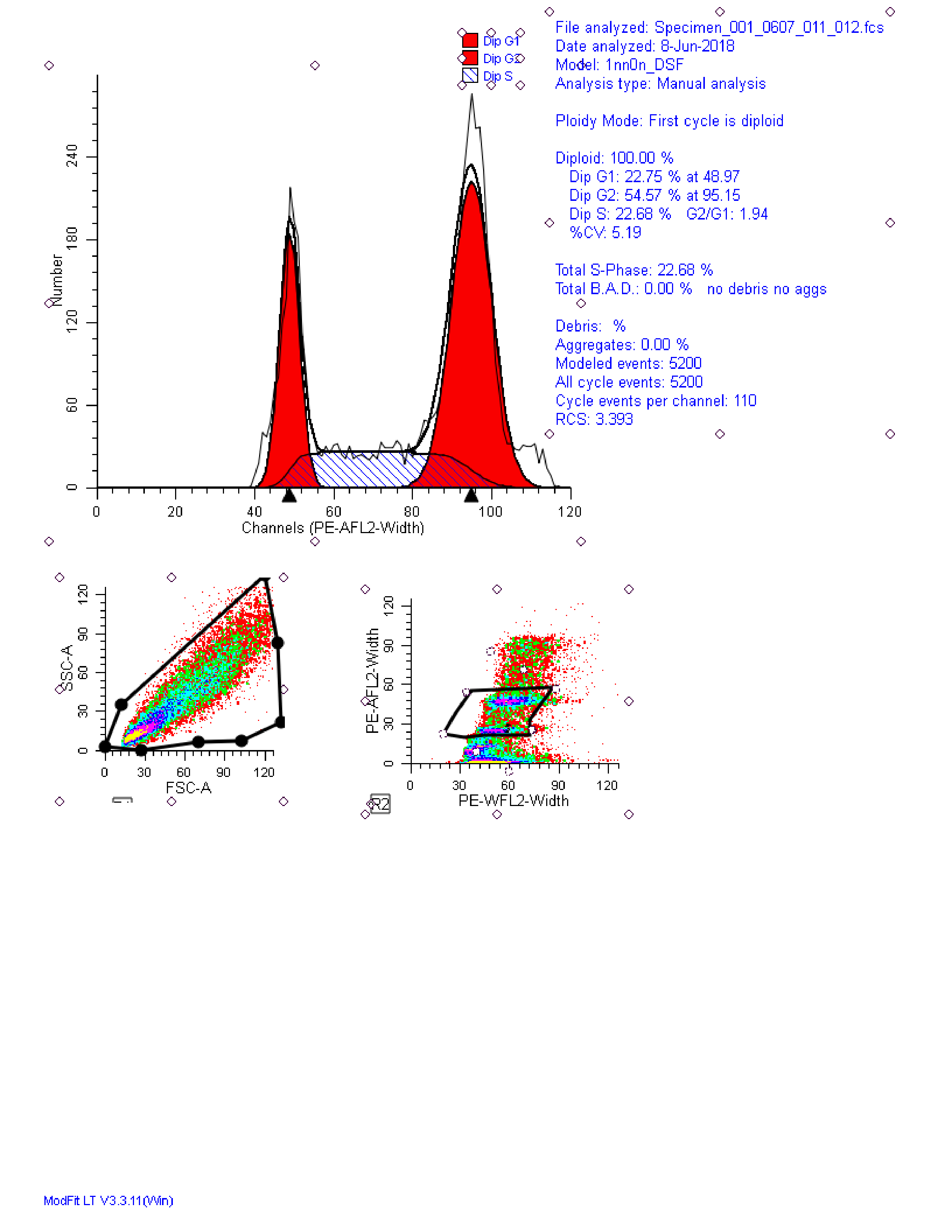

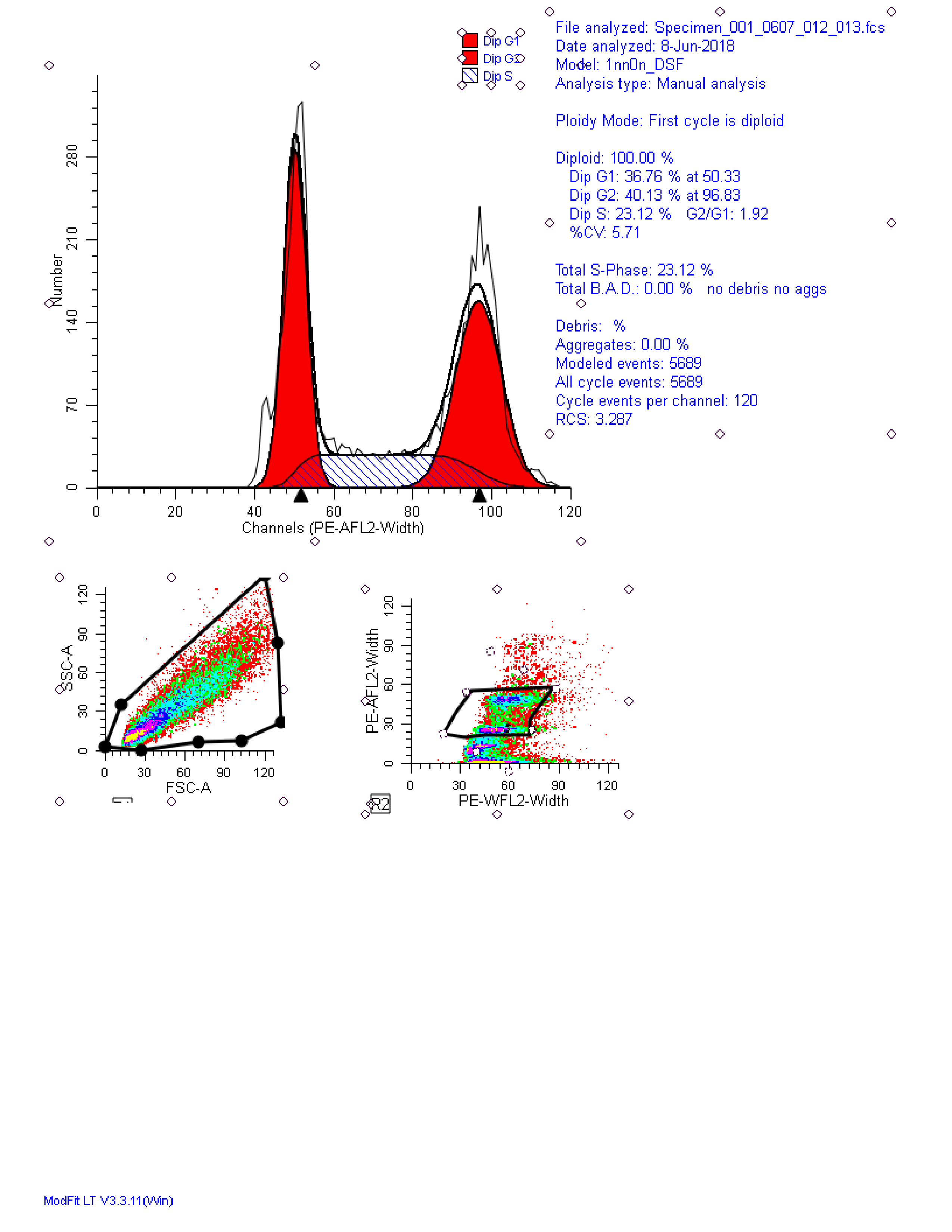


CyclinB1


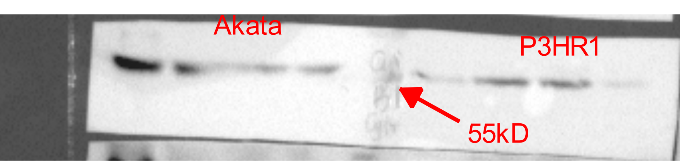


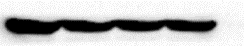


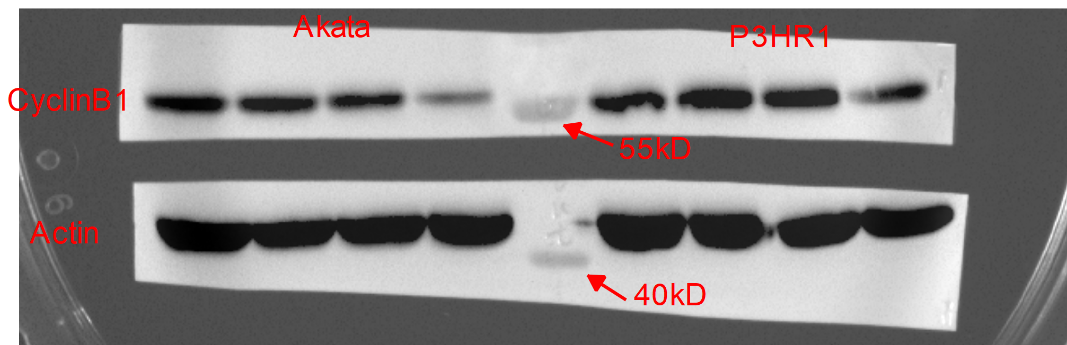

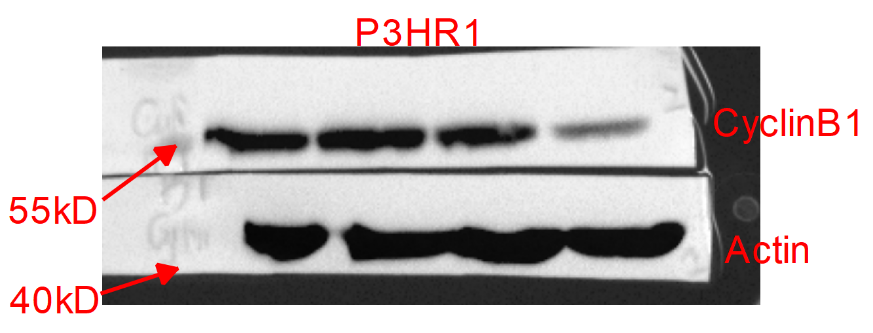

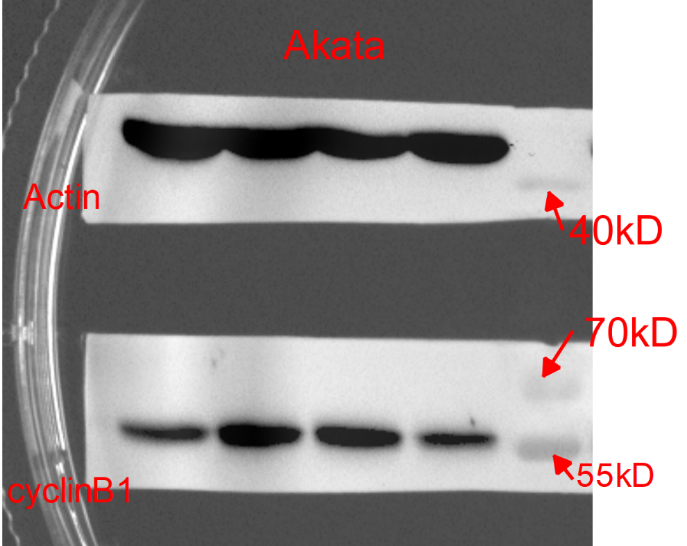
Actin

Supplement: Supplemental Information 5 [file peerj-11-16581-s005.docx]

Figure3

A


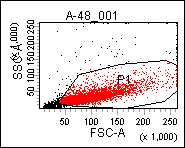

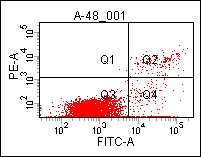

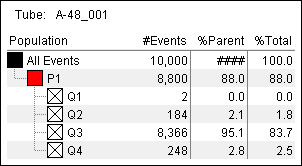

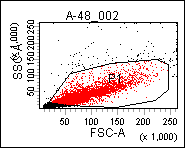

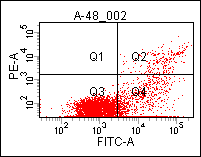

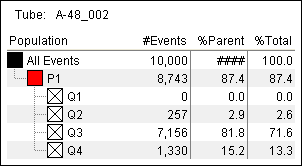

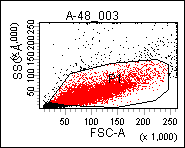

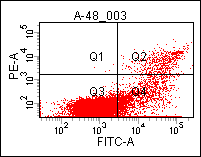

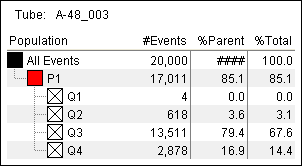

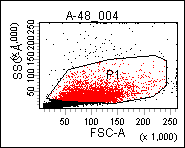

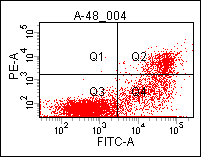

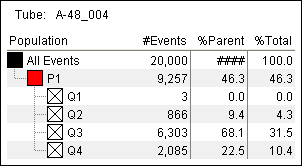


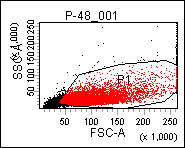

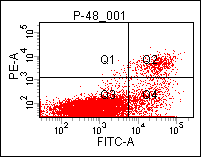

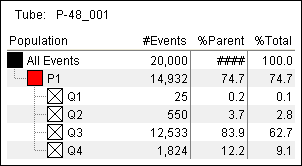

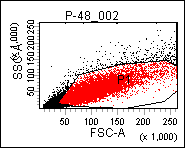

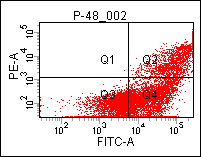

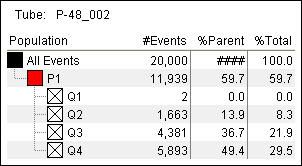

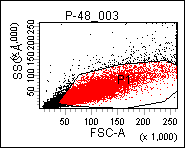

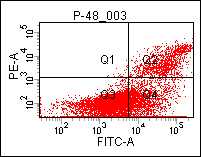

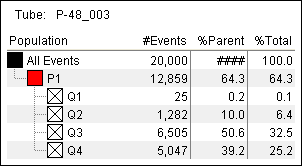

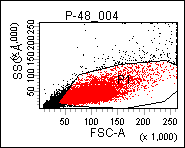

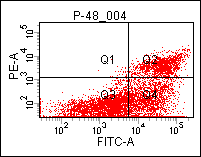

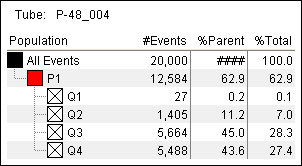


**C**


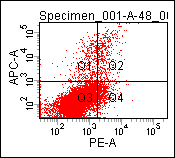

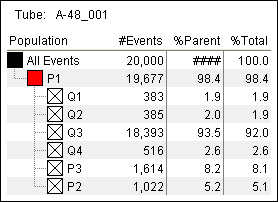


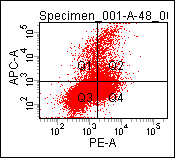

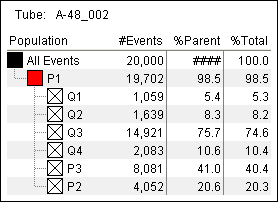


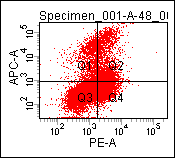

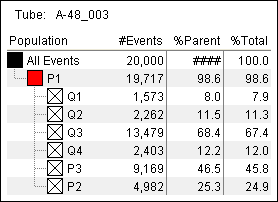


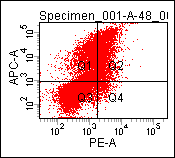

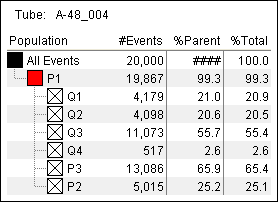


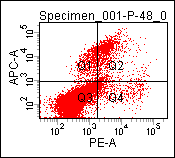

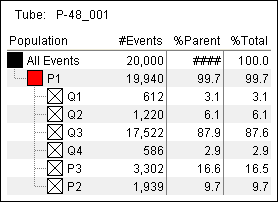


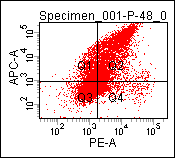

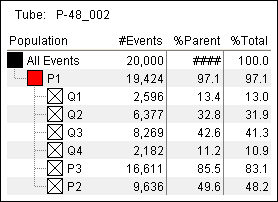


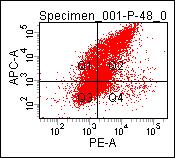

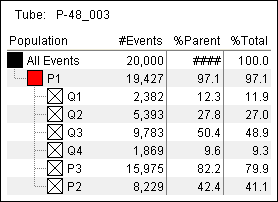


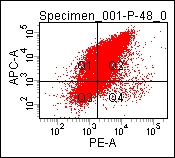

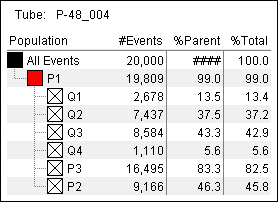


**E** cleaved PARP


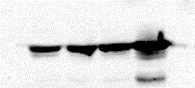
 Akata P3HR1


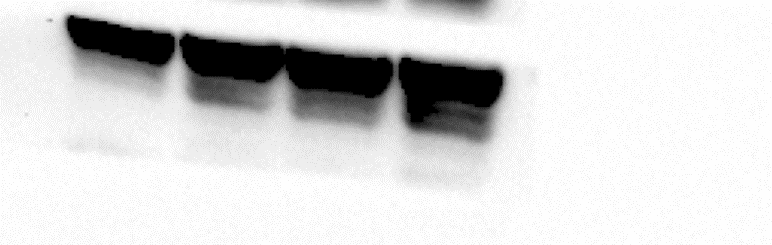


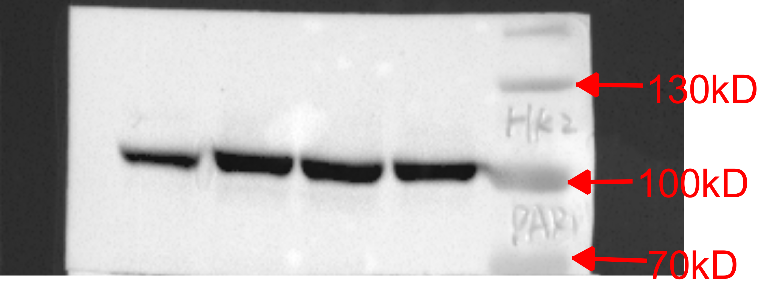

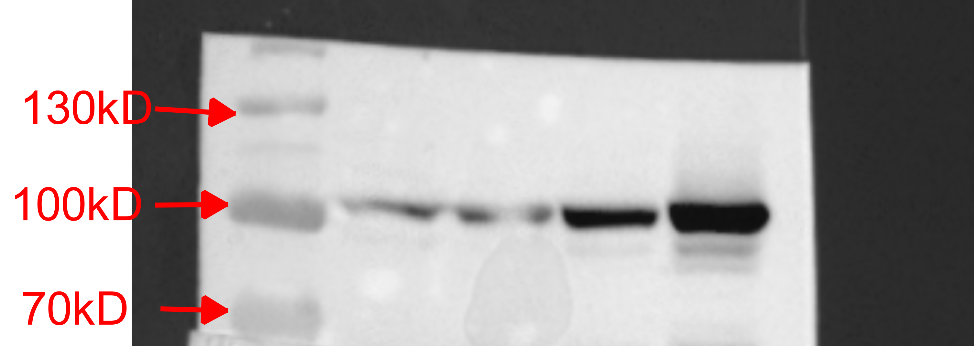


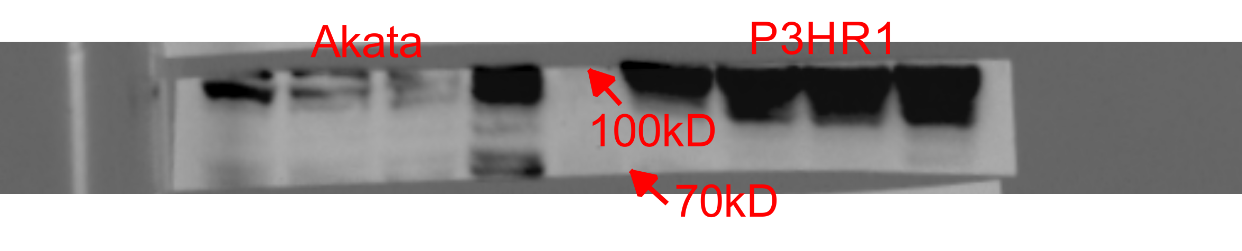


pro-Caspase3

Akata P3HR1


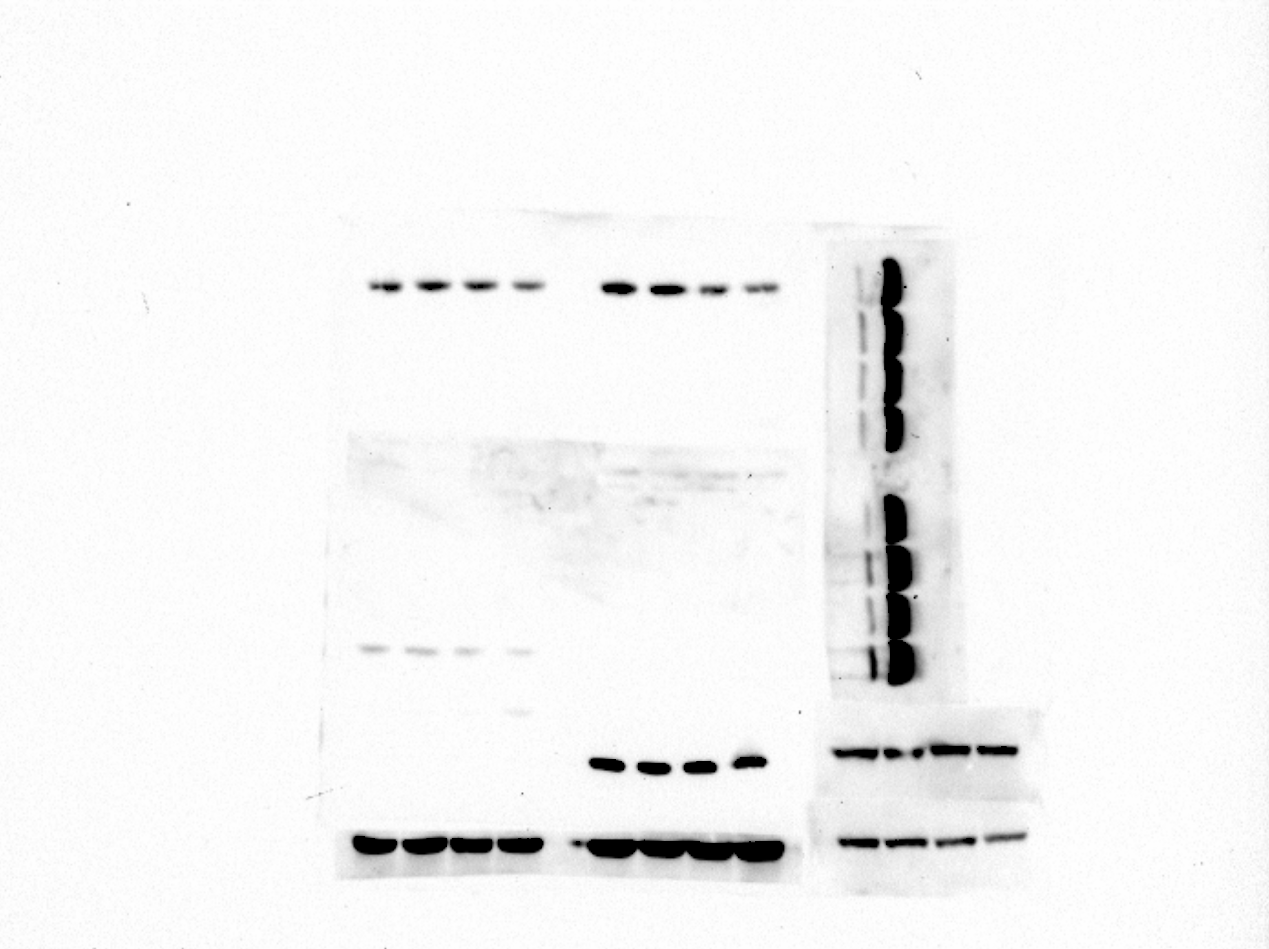


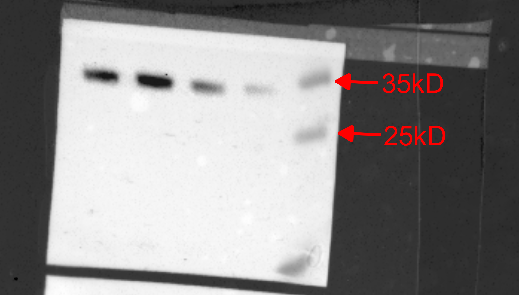


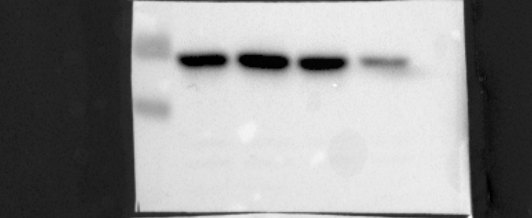


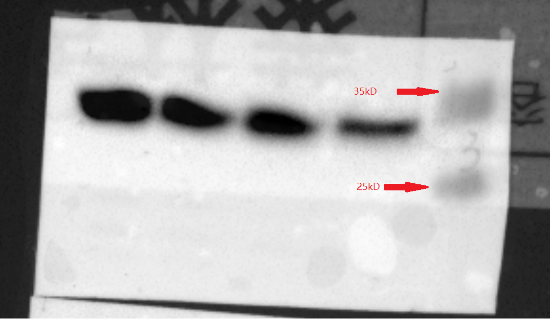


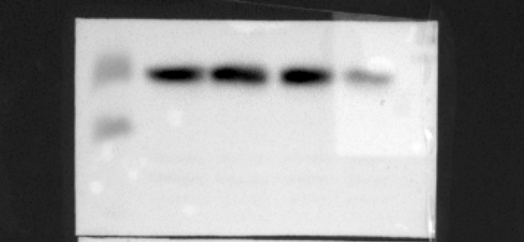


actin

Akata P3HR1


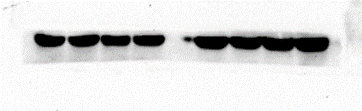


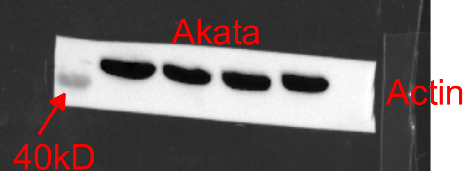

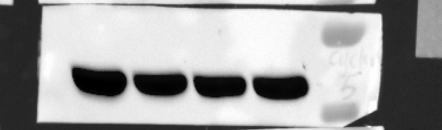

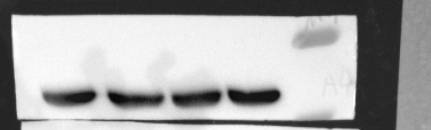

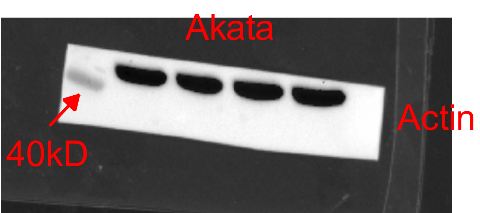

Supplement: Supplemental Information 6 [file peerj-11-16581-s006.docx]

Figure4

A

B


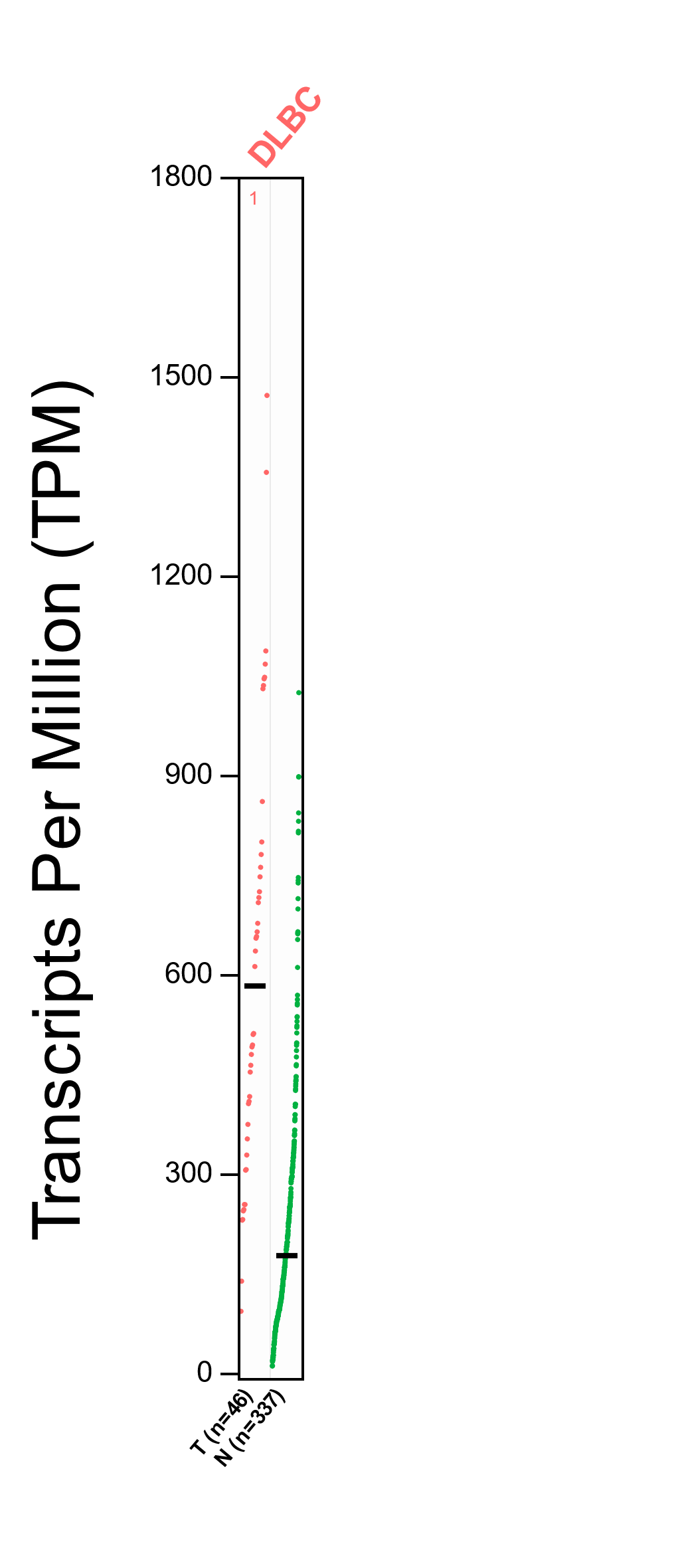

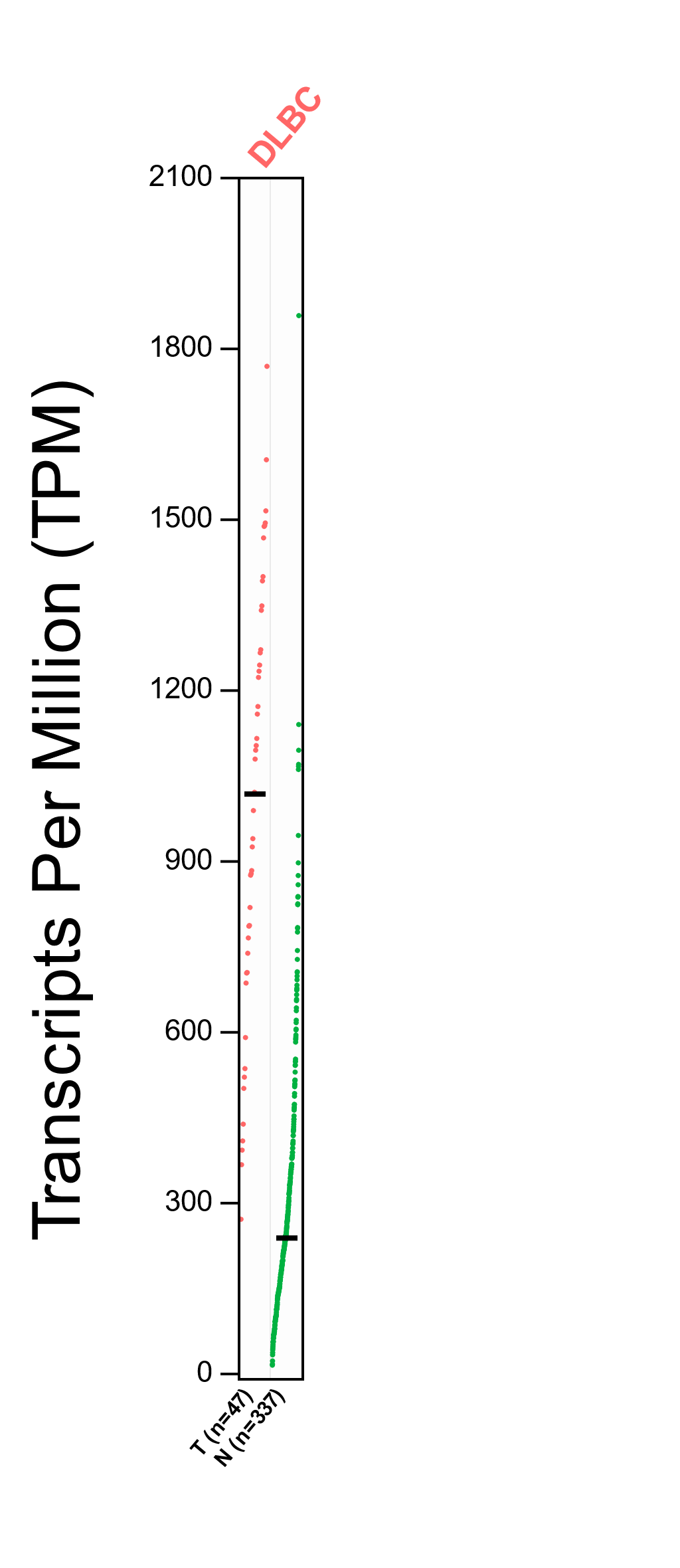

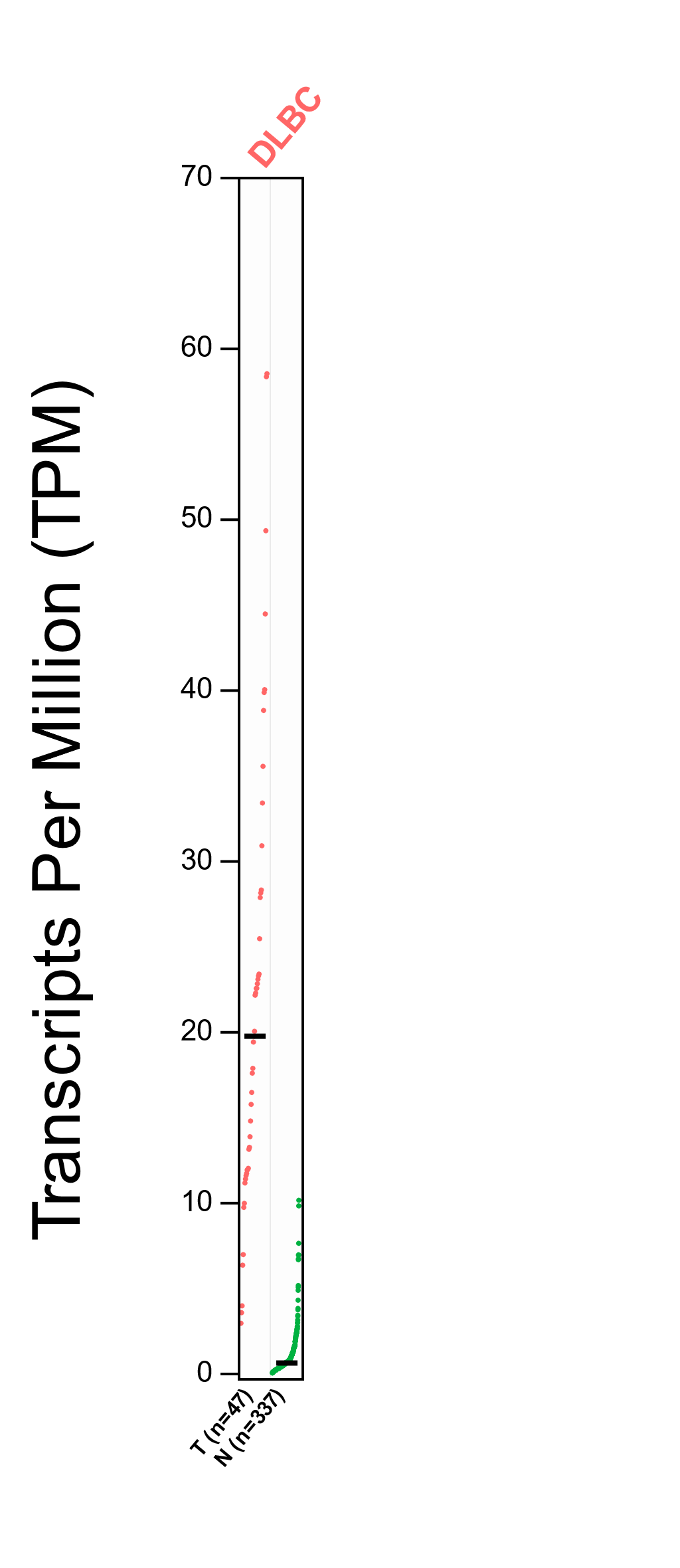

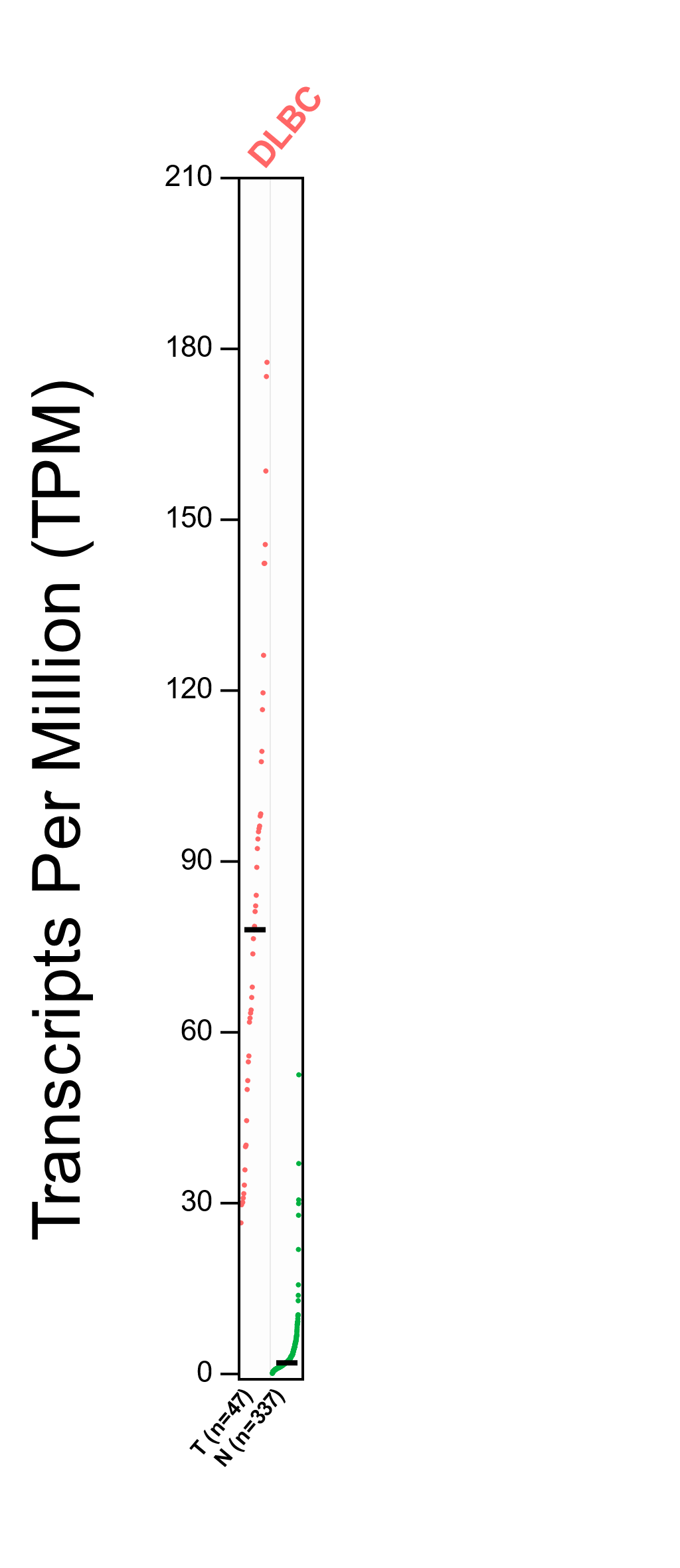


C:

BL-PKM


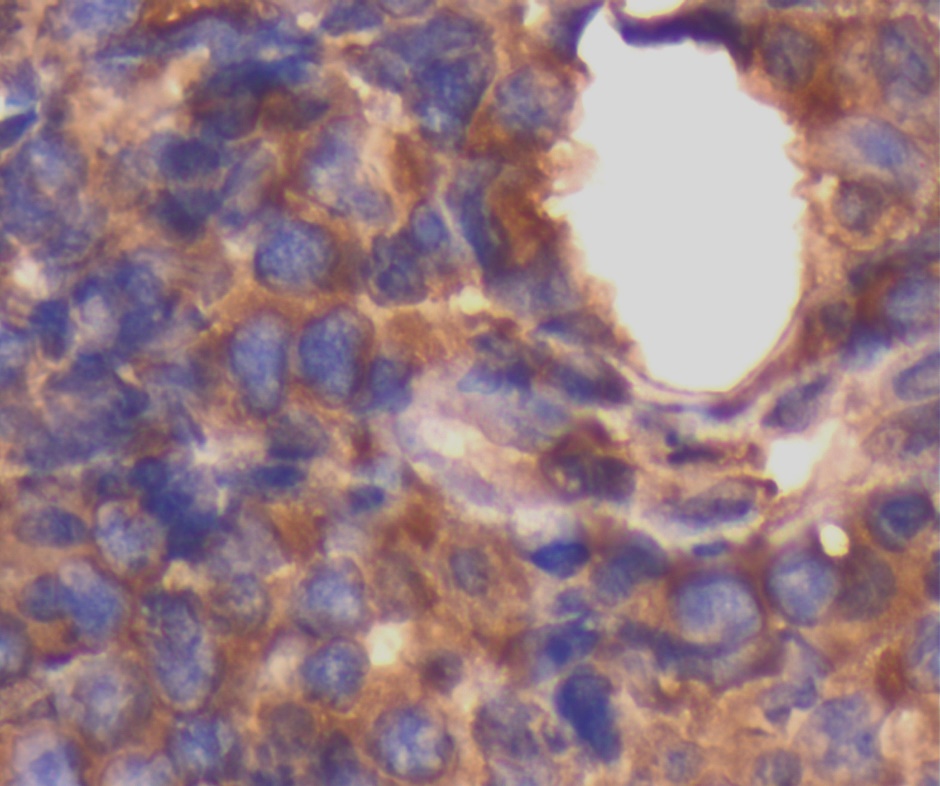


Control PKM


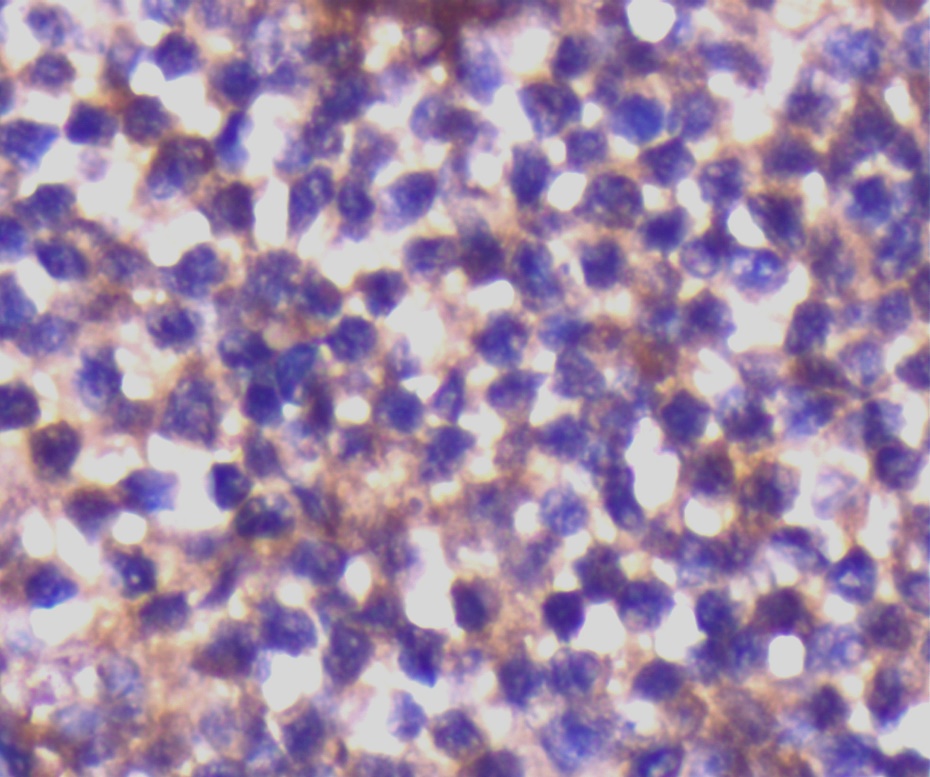


BL-LDHA


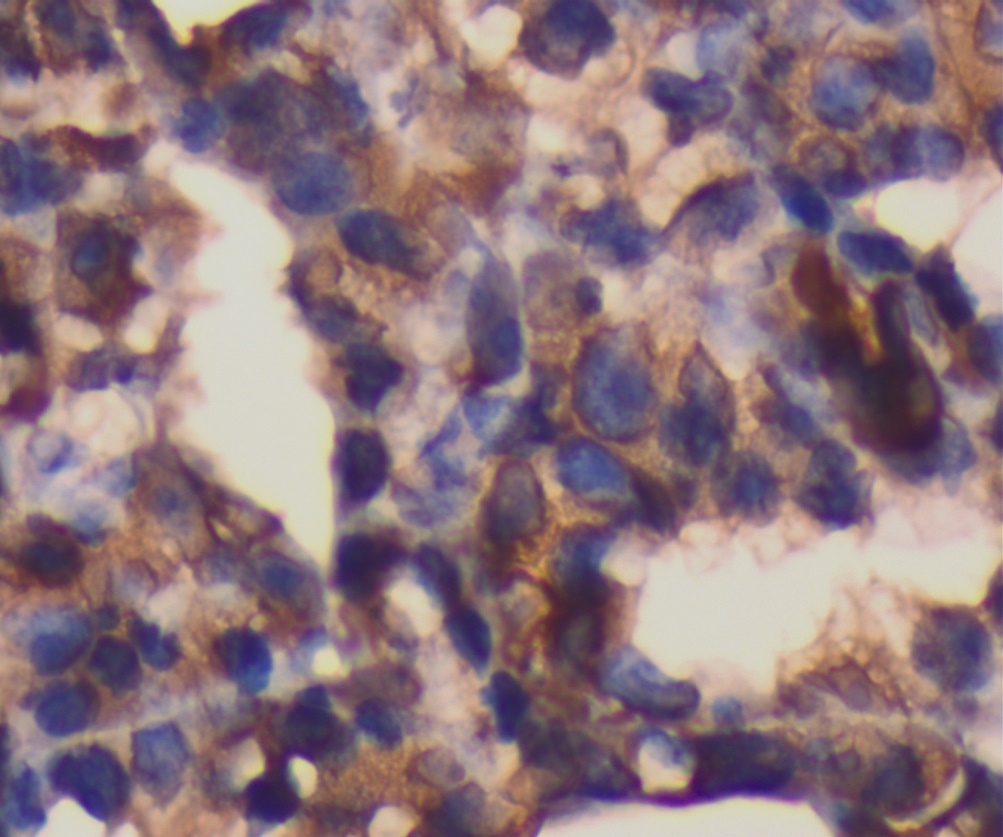


Contol-LDHA


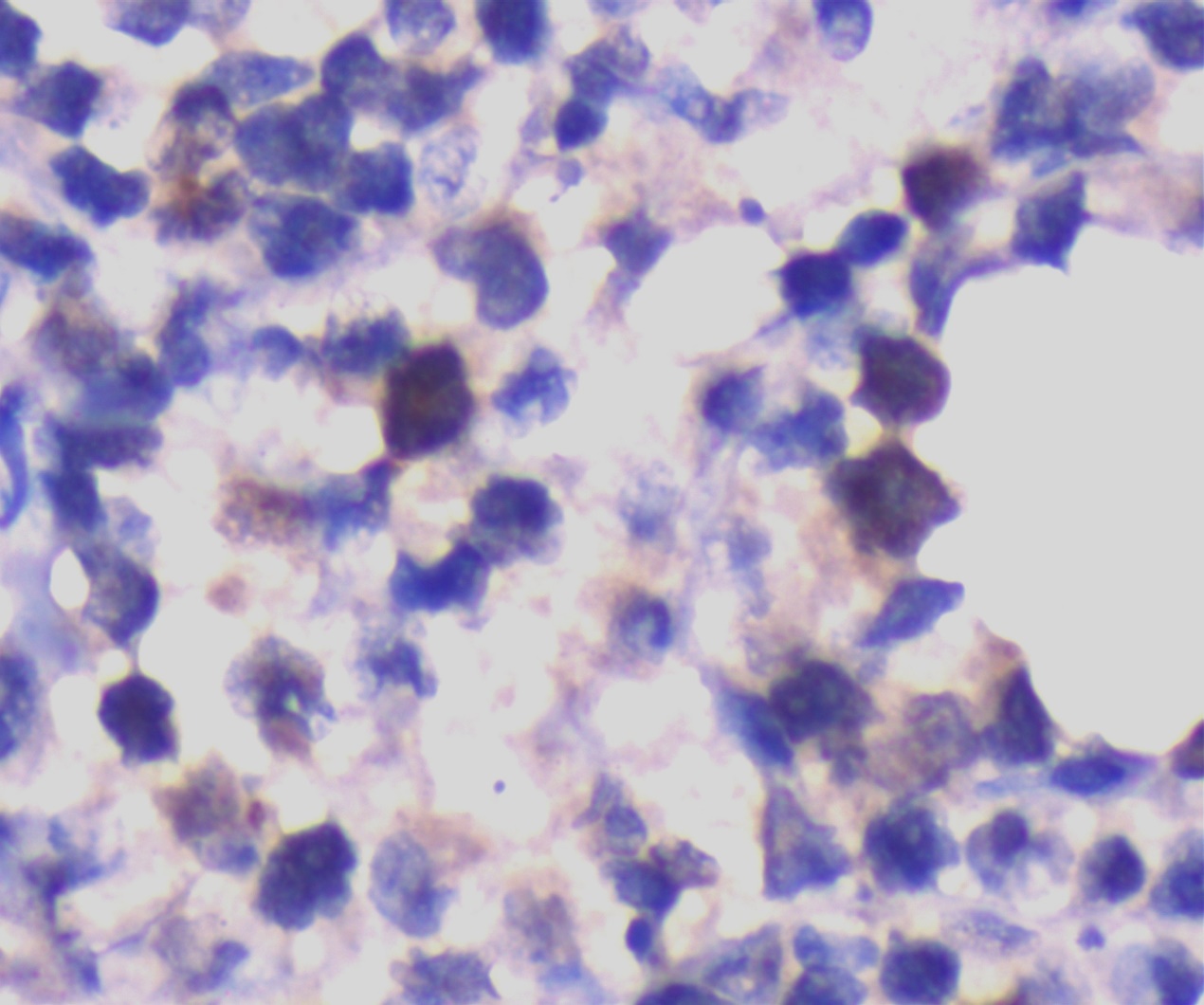

Supplement: Supplemental Information 7 [file peerj-11-16581-s007.docx]

Figure5

C:

Akata P3HR1


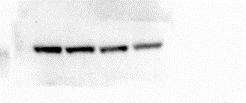

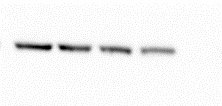
HK2


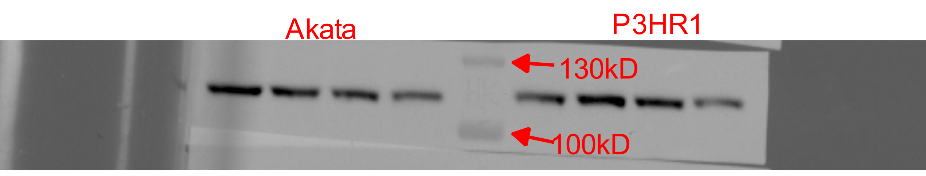


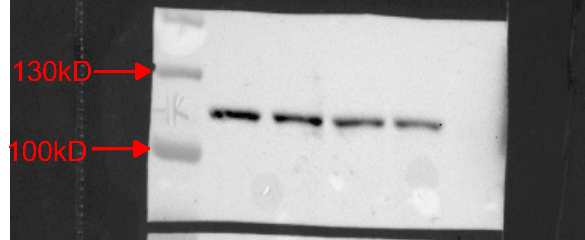


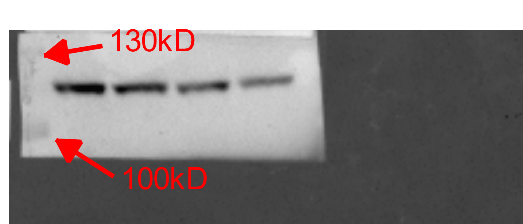


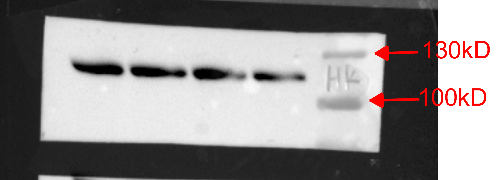


PKM2 Akata P3HR1


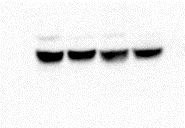


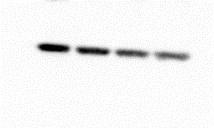


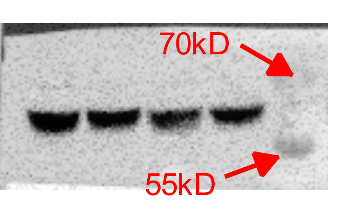


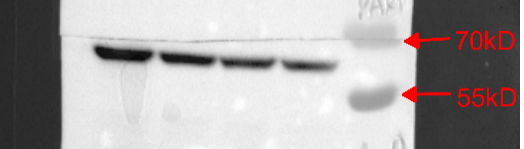


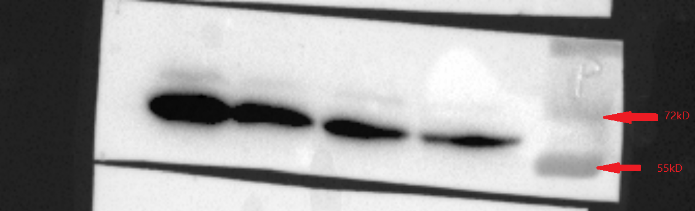

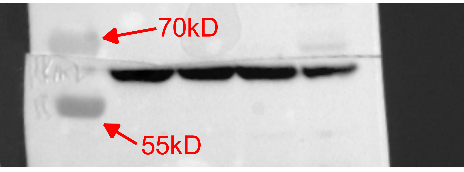


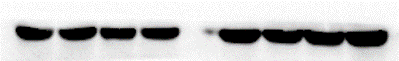
Actin


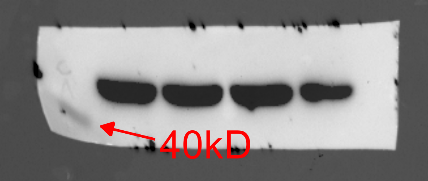

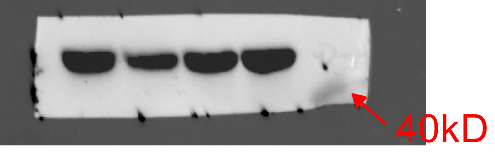


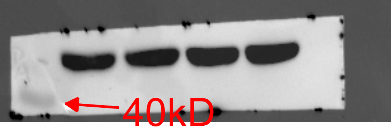

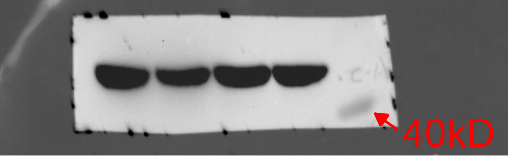

Supplement: Supplemental Information 8 [file peerj-11-16581-s008.docx]

Figure6

A

c-MYC Akata P3HR1


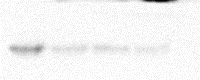


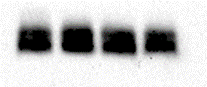


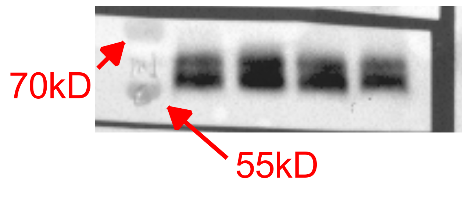

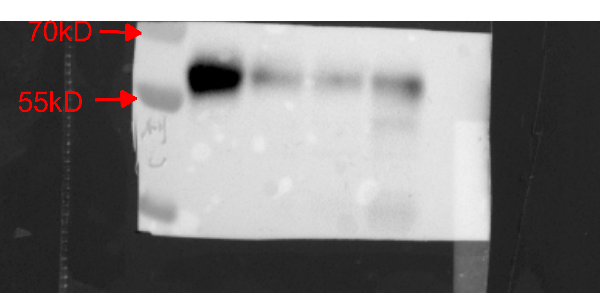


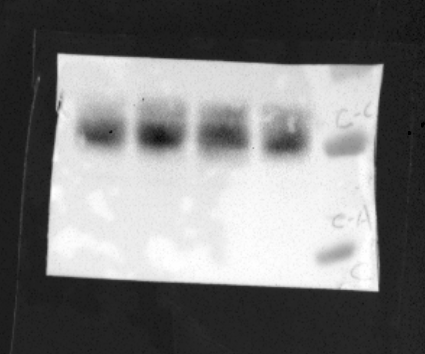


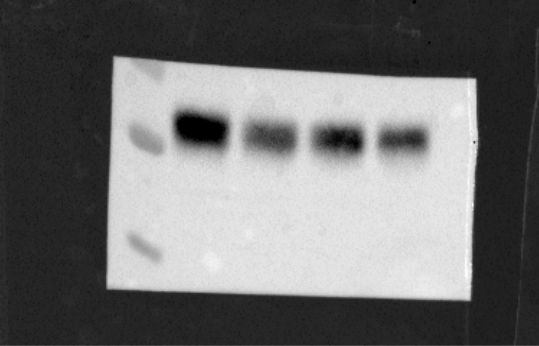


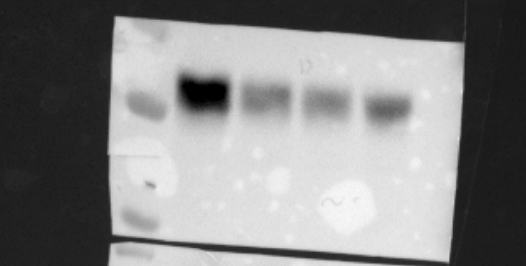


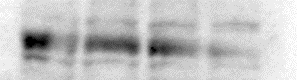

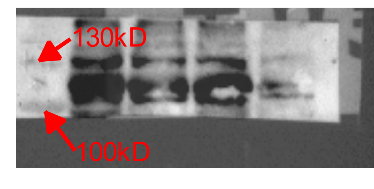
HIF


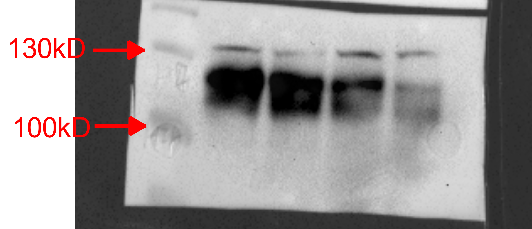


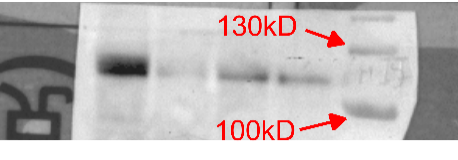


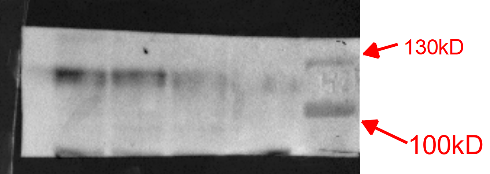


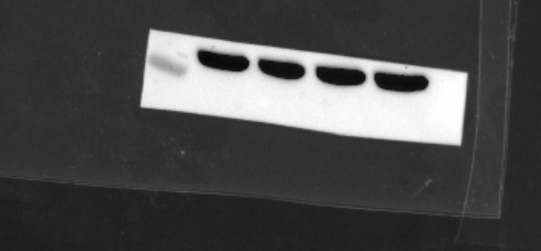

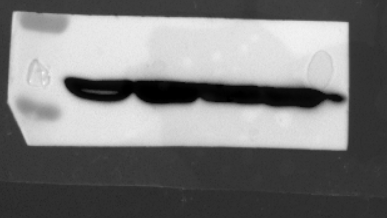

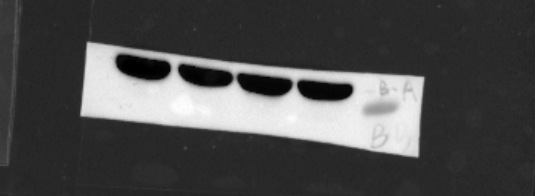

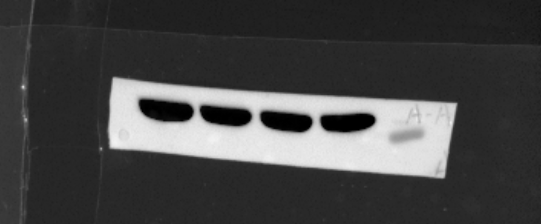

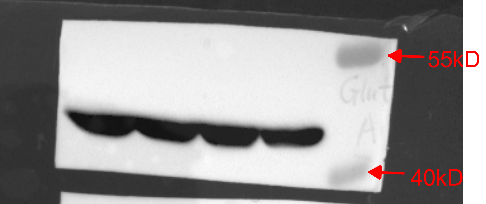

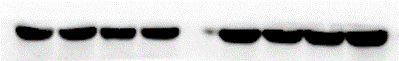

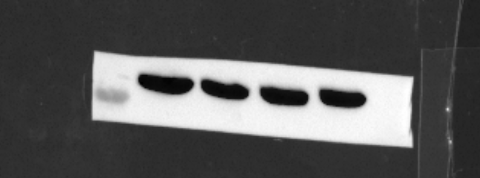
Actin

Supplement: Supplemental Information 9 [file peerj-11-16581-s009.docx]
