# Supplementary material for: The Aurora kinase inhibitor AT9283 inhibits Burkitt lymphoma growth by regulating Warburg effect
Source: PeerJ. 2023 Dec 11;11:e16581. doi: 10.7717/peerj.16581 (PMC10720464; doi:10.7717/peerj.16581)
Supplement: Supplemental Information 12 [file peerj-11-16581-s012.docx]

**Supplementary Information**

**The Aurora Kinase Inhibitor AT9283 Inhibits Burkitt Lymphoma Growth by Regulating Warburg Effect Materials and Methods**

**Quantitative reverse transcription polymerase chain reaction**

Total RNA was extracted from cells using TRIzol reagent (Takara Bio, Inc., Otsu, Japan) according to the manufacturer’s protocols. Primer sequences for messenger RNA (mRNA) are available in the supplementary materials. Complementary DNA was synthesized using 1–5 μg of puriﬁed RNA, the RevertAid™ First Strand cDNA Synthesis Kit (Fermentas, EU) and Oligo (dT). Quantitative reverse transcription PCR (qRT-PCR) was performed on a LightCycler 480 thermal cycler (Roche, Branchburg, NJ). Relative mRNA abundance was calculated using β-actin as an internal control using the 2ΔΔCT method. The mRNA changes are represented relative to untreated cells.

**Table 1.** Sequences of oligonucleotide primers used for RT-qPCR

| Gene | Sequence |
| --- | --- |
| Aurora A | fwd: 5΄- GAGGTCCAAAACGTGTTCTCG -3΄ |
|  | rev: 5΄-ACAGGATGAGGTACACTGGTTG -3΄ |
| Aurora B | fwd: 5΄- CAGAAGAGCTGCACATTTGACG -3΄ |
|  | rev: 5΄-CCTTGAGCCCTAAGAGCAGATTT -3΄ |
| PKM | fwd: 5΄-CCAGCAACGCTTGTAGAACTCA -3΄ |
|  | rev: 5΄-GCTGTCACCCTCTTGCCATCT -3΄ |
| HK2 | fwd: 5΄-AATTGTCCATGTGCTTCCCTA -3΄ |
|  | rev: 5΄-ATCCAGGTTTAATGTCTGTGCTT -3΄ |
| HIFα | fwd: 5΄-ATCCATGTGACCATGAGGAAATG -3΄ |
|  | rev: 5΄-TCGGCTAGTTAGGGTACACTTC -3΄ |
| c-Myc | fwd: 5΄-TCAAGAGGCGAACACACAAC-3΄ |
|  | rev: 5΄-GGCCTTTTCATTGTTTTCCA-3΄ |
| β-actin | fwd: 5΄-TTCCAGCCTTCCTTCCTGGG-3΄ |
|  | rev: 5΄-TTGCGCTCAGGAGGAGCAAT-3΄ |
